# Looking into Working Memory to Verify Potential Search Targets

**DOI:** 10.1523/JNEUROSCI.0091-25.2025

**Published:** 2025-08-20

**Authors:** Sisi Wang (王思思), Freek van Ede

**Affiliations:** Department of Experimental and Applied Psychology, Institute for Brain and Behavior Amsterdam, Vrije Universiteit Amsterdam, Amsterdam 1081 HV, The Netherlands

**Keywords:** internal representation, microsaccades, selective attention, theta oscillations, visual search, working memory

## Abstract

Finding what you are looking for is a ubiquitous task in everyday life that relies on a two-way comparison between what is currently viewed and internal search goals held in memory. Despite a wealth of studies tracking visual verification among external contents of perception, complementary verification processes among internal contents of memory remain elusive. Building on a recently established gaze marker of internal visual focusing in working memory, we uncover the internal inspection process associated with confirming or dismissing potential search targets. We show how male and female human participants “look back” into working memory when faced with external stimuli that are perceived as potential targets and link such internal inspection to the time needed for visual verification. A direct comparison between visual verification among the contents of working memory or perception further revealed how verification in both domains engages frontal theta activity in scalp electroencephalography but also how mnemonic verification is slower to deploy than perceptual verification. This establishes internal verification as an integral component of visual search and provides new ways to look into this underexplored component of human search behavior.

## Significance Statement

Finding what you are looking for is something we engage in all the time and is central to the study of mind, brain, and behavior. To support efficient search, it is well known that we are drawn to inspect those external stimuli that resemble search goals held “in mind.” We now show that search also engages inspection of the internal search goals themselves and link this internal inspection process to the time needed to confirm or dismiss whether we found what we are looking for. Our work puts internal verification behavior on the map as a foundational component of visual search and brings a powerful novel approach to isolate and track such internal verification behavior.

## Introduction

Finding what you are looking for is a ubiquitous task in daily life. Models of visual search naturally invoke a comparison of what is currently viewed to a search “template” in mind (Duncan and Humphreys, 1989; Vickery et al., 2005; Carlisle et al., 2011; Olivers et al., 2011; Eimer, 2014; Myers et al., 2015; Wolfe and Horowitz, 2017; Grubert and Eimer, 2018; Wolfe, 2021). This enables to verify whether something in external view does or does not match one's internal search goal held in working memory.

Visual verification is classically characterized by how we explore and inspect external stimuli: we gravitate toward and inspect external stimuli that resemble what we are looking for (Duncan and Humphreys, 1989; Downing, 2000; Olivers et al., 2006; Avraham et al., 2008; Soto et al., 2008; Becker, 2011; Horstmann et al., 2016; Geng and Witkowski, 2019; Hamblin-Frohman and Becker, 2021). Critically, because visual verification is fundamentally about finding a match between internal and external information, the verification process likely involves a two-way stream: just like internal memory templates prompt inspection of resembling external stimuli, so too may external stimuli prompt inspection of internal search-target representations they resemble to confirm or dismiss a match.

Prior studies have shown that when an external visual stimulus is compared with multiple visual objects in working memory, we return to the matching memory object (Kuo et al., 2009; Dell’Acqua et al., 2010; Theeuwes et al., 2011). Yet, two foundational questions remain unaddressed. First, if this process reflects internal inspection-for-verification, it should not only occur when a visual target matches a mnemonic search goal but also when it resembles what you are looking for. Aforementioned studies did not include resembling potential targets, leaving it ambiguous whether findings reflected the verification process itself (going back in mind to verify) or the outcome of successful verification (going back in mind after a target is verified). Second, this putative internal inspection-for-verification process has yet to be linked to actual verification behavior.

To address these questions, we adapted the classic delayed match-to-sample task (Fuster and Alexander, 1971; Cohen et al., 1997; D’Esposito and Postle, 1999) in which participants judged whether a central stimulus matched either of two visual representations held in working memory. Rather than focusing on the working-memory delay (Todd and Marois, 2004; Vogel and Machizawa, 2004; Sauseng et al., 2009), we used this task to study the core “search process” of inspecting memory contents at test, when verifying a potential match. Using spatially separated memory objects, we could isolate internal visual inspection among the content of working memory (as in Astle et al., 2009; Kuo et al., 2009; Dell’Acqua et al., 2010; Theeuwes et al., 2011; Töllner et al., 2014; van Ede et al., 2020; Wang and van Ede, 2025). We here uniquely tracked such internal inspection through spatial biases in microsaccades that we have recently uncovered as a sensitive marker of internal visual focusing (van Ede et al., 2019, 2020, 2021; Draschkow et al., 2022; Liu et al., 2022; de Vries et al., 2023; Wang and van Ede, 2024, 2025). Critically, by including lures that resembled either memory object, we could study mnemonic verification also following target-resembling lures that we hypothesized to prompt mnemonic inspection.

We show how people “look back” into working memory when faced with external stimuli that are perceived as potential targets and link such internal inspection to the time needed for visual verification. A comparison between visual verification among the contents of working memory or perception further revealed how verification in both domains engages frontal theta activity in scalp electroencephalography (EEG; cf. Jensen and Tesche, 2002; Cavanagh and Frank, 2014; van Ede et al., 2017; Cooper et al., 2019; de Vries et al., 2019; Chidharom and Carlisle, 2023; Wen et al., 2023; Ding et al., 2024) and how mnemonic verification is slower to deploy than perceptual verification.

## Materials and Methods

### Participants

We performed two complementary experiments. Because the main purpose of Experiment 2 was to replicate and extend our core findings from Experiment 1, we exclusively cover Experiment 2-specific methods and results in Text S1. Here, we describe the methods as they apply to Experiment 1.

Twenty-five healthy human volunteers participated in the experiment (ages 18–46 years; M, 24.2; SD, 7.2; 16 females, 9 males). The sample size was determined based on previous studies from our lab with similar experimental designs and similar EEG and eye-tracking outcome variables (van Ede et al., 2019, 2020, 2021; Draschkow et al., 2022; Liu et al., 2022; de Vries et al., 2023; Wang and van Ede 2024, 2025). EEG data from two participants could not be used due to incomplete signal recording. The experimental procedures were reviewed and approved by the Research Ethics Committee of the Faculty of Behavioral and Movement Sciences at the Vrije Universiteit Amsterdam. All participants provided written informed consent prior to the study and were compensated with €10 per hour or equivalent credits.

### Task and procedure

**Figure 1.**
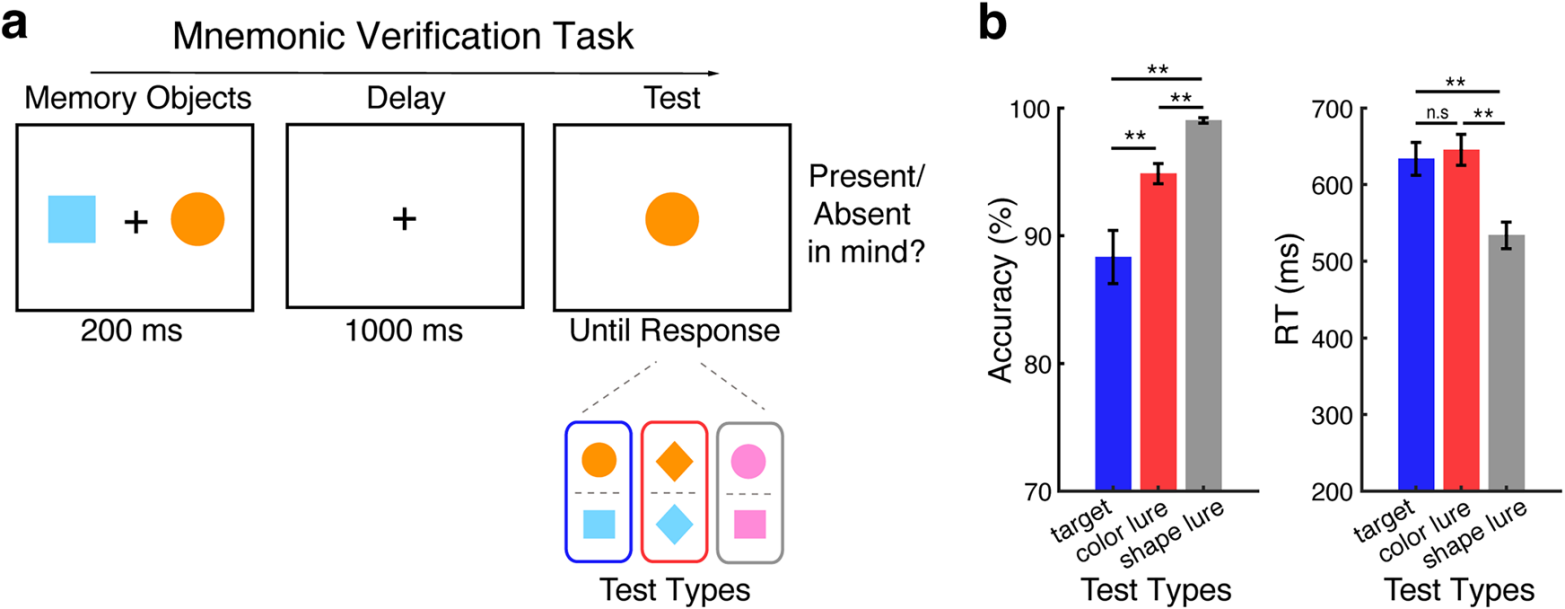
Visual target verification depends on perceived similarity between memorized and perceived objects. ***a***, Top panel, Schematic of the mnemonic-verification task. Participants memorized two visual objects (encoded to the left and right of fixation) and reported whether a subsequent centrally presented test stimulus was identical to either memory object (i.e., present/absent). Bottom panel, Possible test-stimulus types. Test stimuli could be actual targets (color and shape match with one of the memory objects, as illustrated in the blue frame), color lures (only color match with one of the memory objects, as illustrated in the red frame), or shape lures (only shape match with one of the memory objects, as illustrated in the gray frame). Color and shape lures required an “absent” report. Targets and lures only matches one or both features with one of the memory objects (i.e., the left or the right object) and never with the other. ***b***, Response accuracy (left panel) and response time (right panel) across test types. Error bars indicate ±1 SEM. ** represents *p* < 0.001; n.s represents not significant.

Human observers completed two versions of a visual-verification task within a single experiment: one requiring verification of whether a visual stimulus on the screen matched either of two internal visual representations held in working memory (“mnemonic verification”; Fig. 1*a*) and the other requiring verification of whether a single visual representation in working memory matched either of the two external visual stimuli on the screen (“perceptual verification”; Fig. 4*a*; akin to the approach in Kuo et al., 2009; Dell’Acqua et al., 2010). The key distinction between the mnemonic and perceptual-verification tasks lies in whether participants are comparing a single centrally presented visual target among two mnemonic representations held in working memory (enabling us to use spatial markers to isolate and track internal inspection of the left/right memory object) or comparing a single centrally encoded mnemonic target against two visual stimuli presented on the screen (enabling us to use spatial markers to isolate and track external inspection of the left/right perceptual object).

In the mnemonic-verification task, each trial (Fig. 1*a*) began with a memory array displaying two objects (each with distinct colors and shapes) on the left and right sides of the screen for 200 ms. This was followed by a 1,000 ms delay period during which only the central fixation cross remained on the screen. Next, a memory test was presented at the center of the screen. Participants were instructed to respond whether the test object was identical (same color and shape) to one of the memorized objects (“same”) or different from both (“different”). The test object was chosen from the following three types with equal probability: a “target” that matched one of the memorized objects, a “color lure” that matched the color of one of the memorized objects but had a different shape, or a “shape lure” that matched the shape of one of the memorized objects but had a different color. Of the two memory objects, there was always one object that would either be a full match or a lure that matched only in color or only in shape. The other memory object was always chosen such that it did not match in shape nor in color, thus serving as the neutral object. The logic was as follows: if mnemonic verification engages visual inspection of the potential memory match (be it a target or lure), we could track this in spatial attentional deployment to the memorized location of the target or lure memory object. A “same” response was only correct for the “target” test. In trials with only a lure, a “different” response was the correct answer. The location of the target/lure and neutral memory object were counterbalanced across trials.

A critical difference between our current mnemonic-verification task and prior “retrocue” tasks is that while in retrocue tasks the retrocue explicitly instructs to shift attention to the relevant memory content, in our mnemonic-verification task, we never explicitly asked participants to attend to specific content in memory. Our central question is, in fact, whether the visual verification processes engaged in our task “do” or “do not” trigger internal attention shifts, akin to those observed following explicit retrocues. Specifically, we here build on our prior work establishing spatial biases in microsaccades as a reliable marker of internal attention shifts in working memory (when prompted to shift attention; van Ede et al., 2019; 2020, 2021; Draschkow et al., 2022; Liu et al., 2022; de Vries et al., 2023; Wang and van Ede 2024, 2025) and now leverage this established link to use this marker to ask when and whether such attention shifts also occur as a natural part of visual verification.

The trial procedure for the complementary perceptual-verification version of our task closely mirrors that of the mnemonic-verification version, with the main difference being the reversal of the memory and test array sequence (Fig. 4*a*). In the perceptual-verification task, participants first viewed a single memory object (search template) presented centrally. They then responded to whether this memorized object was present or absent among two test objects displayed on the left and right sides of the screen that would again contain either a target or lure on the one side and a neutral object on the other side.

In both tasks, participants pressed either the “F” or “J” key on the keyboard for the “same” and “different” (or “present” and “absent”) responses, with the key–response association counterbalanced across participants. Feedback was provided after each response, with “1” indicating a correct answer and “x” indicating an incorrect answer.

Each participant completed four sessions, with each session comprising 432 trials—yielding a total of 1,728 trials per participant. Two out of four sessions involved the mnemonic-verification version of the task, while the other two sessions involved the perceptual-verification version of the task. The order of tasks was pseudorandomized across participants. In both tasks, one-third of trials contained a target, another third of trials contained a color lure, and the final third of trials contained a shape lure. We intentionally designed the experiment this way to balance the number of trials across the three test conditions, thereby making our dependent variables comparable across conditions, at least in terms of trial numbers. This resulted in 288 trials per condition, with a total of six conditions (2 verification tasks × 3 test types). Prior to the first session, participants practiced 18 trials covering all possible conditions. The entire experiment lasted ∼2 h.

### Apparatus and stimuli

Stimuli were presented using MATLAB (R2022a; MathWorks) and the Psychophysics Toolbox (version 3.0.16; Brainard, 1997) on a 23 inch LED monitor with a resolution of 1,920 by 1,080 pixels, operating at a 240 Hz refresh rate. Participants were seated 70 cm from the screen, with their heads supported by a chin rest to ensure stable eye-tracking.

A gray background (RGB, 192, 192, 192) was used throughout the experiment, with a central fixation cross (0.75° in width and height) displayed at the center of the screen. Both memory and test objects consisted of colored shapes, randomly selected from three shapes of equal area [square (1.2° side length), circle (1.36° diameter), or diamond (1.2° side length)] and three colors [blue (RGB, 102, 255, 255), orange (RGB, 255, 153, 51), or pink (RGB, 255, 102, 255)]. The two memory objects (in the mnemonic-verification task) or visual objects (in the perceptual-verification task) were displayed 5.4° to the left and right of central fixation, each with a unique shape and color.

#### Analysis of behavioral data

 Response accuracy was calculated as the proportion of correct responses. Reaction time (RT) was measured as the interval from the onset of the memory test to the completion of the response. Trials with RTs exceeding 2 s were excluded from subsequent analysis, retaining 99.44% (SD, 0.7%) of trials for further analysis.

#### Eye-tracking acquisition and preprocessing

 The eye tracker (EyeLink 1000, SR Research) was positioned ∼5 cm in front of the monitor, on a table ∼65 cm away from the participants' eyes. Horizontal and vertical gaze positions were recorded for a single eye at a sampling rate of 1,000 Hz. Prior to recording, the eye tracker was calibrated using the built-in calibration and validation protocols of the EyeLink software. Participants were instructed to rest their chin on the chin rest for the entire duration of each experimental block following calibration.

Offline, eye-tracking data were converted from the .edf format to .asc format and imported into MATLAB using FieldTrip (Oostenveld et al., 2011). Blinks were identified by detecting clusters of zeros in the eye-tracking data. Data from 100 ms before to 100 ms after each detected blink cluster were marked as Not-a-Number (NaN) to exclude residual blink artifacts. Following blink correction, the data were epoched from −200 ms to 1,000 ms relative to the onset of the memory test.

### Saccade detection

For saccade detection, we utilized a velocity-based approach [cf. Engbert and Kliegl, 2003 that followed the procedure described in Liu et al. (2022)] and that we successfully employed in several prior working-memory studies from our lab (Liu et al., 2022; de Vries et al., 2023; Liu et al., 2024; Wang and van Ede, 2024, 2025). Given that the memory objects in the mnemonic-verification task and the test objects in the perceptual-verification task were always presented horizontally (i.e., center-left and center-right), our saccade detection focused specifically on the horizontal channel of the eye-tracking data (as in Liu et al., 2022; de Vries et al., 2023; Wang and van Ede, 2024, 2025).

We first computed gaze velocity by measuring the distance between consecutive gaze positions over time. To improve precision and reduce noise, we applied temporal smoothing to the velocity data using a Gaussian-weighted moving average filter with a 7 ms sliding window, implemented via MATLAB's “smoothdata” function. The saccade onset was identified as the first instance where the velocity exceeded a trial-specific threshold, set at five times the median velocity. To avoid multiple detections of the same saccade, we imposed a minimum interval of 100 ms between successive saccades.

Saccade magnitude and direction were assessed by comparing gaze positions before the saccade (from −50 to 0 ms before threshold crossing) with positions after the saccade (from 50 to 100 ms after threshold crossing). Each saccade was categorized as “toward” or “away” based on its direction (left/right) and its relation to the location of the memorized object (in the mnemonic-verification task) or the search object (in the perceptual-verification task). A “toward” saccade was aligned with the location of the target or lure-matching object, while an “away” saccade was directed in the opposite direction (toward the neutral object).

After identifying and classifying the saccades, we quantified saccade rates (in hertz) using a sliding time window of 50 ms, advances in steps of 1 ms. For the primary analysis, we focused on the spatial bias in saccades, calculated as the difference in rates (in hertz) between toward and away saccades. Finally, to characterize the nature of the saccades contributing to our reported spatial modulations, we iteratively performed the above analysis as a function of saccade size (as in Liu et al., 2022; de Vries et al., 2023; Liu et al., 2024; Wang and van Ede, 2024, 2025), considering saccade size bins of 0.5° wide, sampling successive bins in steps of 0.1°.

### Saccade bias as a function of verification time

To determine if the observed saccade bias is more pronounced in trials where additional verification is required, we split trials into “fast-verification” and “slow-verification” trials using a median split of RTs. Our median-split analyses (as well as the complementary and more continuous multiple-split analyses that we return to below) were performed across trials within each participant, before averaging outcomes across participants. We did this separately for each condition, using condition-specific medians. This enabled us to compare the saccade bias (toward minus away) between fast and slow-RT trials, separately for the different test types (target, color lure, shape lure) and tasks (mnemonic verification, perceptual verification). The saccade-bias comparison between slow- and fast-RT trials in the current study included only correct-response trials, thereby excluding the possibility that differences in saccade-bias patterns are driven by variations in response accuracy.

We used median splits because the continuous gaze-bias measure of interest required aggregating across multiple trials. Our gaze bias is defined as the rate of saccades directed toward versus away from the test-matching memory object, necessitating the aggregation of multiple trials where the test stimulus matched the shape and/or color of either the left or right memory object. Aggregating across trials further allowed us to construct the continuous gaze-bias time course that was the primary dependent variable of interest. The median-split method further allowed us to visualize the resulting gaze-bias time courses separately for each split—an important step in distinguishing between relevant variability in amplitude and variability in latency. For completeness, we also conducted quartile splits alongside this binary categorization.

### EEG acquisition and preprocessing

EEG signals were recorded using a 64-channel Biosemi system with a sampling rate of 1,024 Hz. Active electrodes were positioned according to the international 10–20 system. The CMS and DRL electrodes, located on the left and right sides of POz, respectively, served as the online reference. Offline, the signals were rereferenced to the average of both mastoids.

To monitor and correct for eye-movement and blink artifacts, we placed two external electrodes horizontally adjacent to the left and right eyes and two electrodes above and below the right eye. These electrooculogram measurements were utilized solely for data cleaning through independent component analysis (ICA), as described below. Additionally, we used an EyeLink system to collect eye-tracking data, which was used to extract our primary eye-tracking outcome measures.

Offline data analyses were performed in MATLAB using a combination of FieldTrip (Oostenveld et al., 2011) and a custom code. After rereferencing, the continuous EEG data were segmented into epochs from −200 to +600 ms relative to the onset of the memory test (as in Nobre et al., 2008; Kuo et al., 2009; Dell’Acqua et al., 2010). We then applied fast ICA using FieldTrip to the (concatenated) EEG epochs to identify and remove components related to blinks and eye movements. ICA components associated with eyeblinks and horizontal eye movements were excluded based on their correlations with vertical electrooculography and horizontal electrooculography signals, respectively. Additionally, we used the “ft_rejectvisual.m” function in FieldTrip with the “summary” method to visually inspect and remove trials with exceptionally high variance, which were considered artifact trials. We did this without knowledge of the condition to which individual trials belonged. After trial removal, we retained 96.9% (SD, 1.97%) of the trials for further EEG data analysis.

### EEG time–frequency analysis

For time–frequency decomposition of the EEG data, we first applied a surface Laplacian transform to enhance the spatial resolution (Babiloni et al., 2001; Carvalhaes and De Barros, 2015; Kayser and Tenke, 2015). Following this, we decomposed the cleaned EEG epochs into time–frequency representations using a short-time Fourier transform with Hanning-tapered data, as implemented in FieldTrip with the “ft_freqanalysis.m” function. A 300 ms sliding time window was used to estimate spectral power in the frequency range of 2–40 Hz, with frequency steps of 1 Hz and time steps of 10 ms.

For the analysis of frontal theta activity, we focused on activity over frontal–central electrodes where ample prior studies have reported frontal theta activity that had been implicated in working memory and cognitive control (Jensen and Tesche, 2002; Cavanagh and Frank, 2014; van Ede et al., 2017; Cooper et al., 2019; de Vries et al., 2019; Chidharom and Carlisle, 2023; Wen et al., 2023; Ding et al., 2024). Inspired by these prior findings of theta activity in frontal–central electrodes, we focused our analysis on time–frequency activity extracted from frontal–central electrode FCz together with its two adjacent neighbors FC1 and FC2.

Prompted by our behavioral and gaze data that indicated robust visual inspection-for-verification for actual targets and color lures but not for shape lures—we leveraged our shape lure condition as a neutral reference condition for our EEG comparisons. This enabled us to contrast the target and color-lure conditions against this condition. We then computed the relative percentage change in time–frequency power in the following ways. For comparisons between target tests and shape lures: [(target tests − shape lures) / (target tests + shape lures)] × 100. For comparisons between color lures and shape lures: [(color lures − shape lures) / (color lures + shape lures)] × 100. In addition, to evaluate whether theta activity is more prominent in trials where additional verification is required, we additionally performed a median split on RTs within each condition to separate fast- and slow-verification trials. We then calculated the percentage change in time–frequency power between slow and fast-RT trials using the formula: [(slow RT − fast RT) / (slow RT + fast RT)] × 100.

Finally, to visualize topographical maps of the relative change in time–frequency power, we averaged data over the critical theta frequency band between 3 and 7 Hz and the time window of 200–600 ms (for the comparison across test types) or 400–600 ms (for the comparison between fast- and slow-verification trials) for all electrodes. Topographies merely served as an additional visualization to verify the frontal nature of the observed activity differences and were not subjected to further statistical testing.

Next to frontal–central theta activity, we also investigated the spatial modulation of posterior 8–12 Hz alpha-band activity following the procedure as in Wang and van Ede (2025). We contrasted time–frequency activity in electrodes P1/2, P3/4, P5/6, P7/8, P9/10, PO3/4, PO7/8, and O1/2 between trials where the feature-matching memory object was contralateral versus ipsilateral to the electrode. We did this separately for targets, color lures, and shape lures.

### Experimental design and statistical analyses

 Participants completed two versions of a visual-verification task (mnemonic version or perceptual version). The tasks contained three key conditions: actual targets, color lures, and shape lures that were intermixed across trials in a within-participant manipulation. Another key manipulation was whether the target/lure object overlapped with the left/right memory object (mnemonic verification) or the left/right test object (perceptual verification).

#### Behavioral performance data

For statistical analysis of the behavioral performance data, we used repeated-measure ANOVAs to assess response accuracy and RTs across the three test types (target test, color lures, and shape lures). We did this separately for accuracy and for RT. Paired-sample *t* tests were performed for post hoc comparisons between each pair of test-type conditions. Bonferroni’s correction method was used to adjust for multiple comparisons, and all reported *p* values are Bonferroni corrected.

#### Saccade bias

To statistically analyze the temporal profiles of the spatial modulations in saccade rates (between toward and away saccades) following the onset of the test display, we utilized a cluster-based permutation approach (Maris and Oostenveld, 2007) with the “ft_timelockstatistics” function in Fieldtrip, employing the Monte Carlo method. This approach is effective for assessing data consistency across multiple adjacent time points while controlling for (i.e., bypassing) multiple comparisons. We performed 10,000 permutations to generate a single permutation distribution of the largest cluster that could occur by chance, to compare the observed clusters in the nonpermuted data against. We identified clusters using Fieldtrip's default settings, which group temporally contiguous data points showing statistical significance (*p* < 0.05) in a mass-univariate *t* test. The cluster size was defined as the sum of *t* values within each cluster. This method was used to evaluate the time-series data of spatial saccade bias against zero and to compare spatial saccade bias between different test types and RT categories (slow- vs fast-RT trials).

In addition, to compare the latency of the observed spatial saccade biases between the mnemonic- and the perceptual-verification versions of the task, we normalized the saccade bias for each version of the task by dividing the observed saccade bias at each time point by the peak value of the saccade bias within that task. We then used a simplified jackknife method [as described in Smulders (2010)] to compare the onset latency of the normalized saccade bias. Onset latency was defined as the time point at which the amplitude of saccade bias reached 50% of the peak value. Paired-sample *t* tests were conducted to assess differences in latency between the two versions of the task.

#### Frontal midline theta

Statistical analysis of the EEG time–frequency employed a similar cluster-based permutation approach as in saccade-bias analyses, with this time clustering being applied in two dimensions: time and frequency. This method was used to compare the relative percentage change of time–frequency power across different test types (e.g., target vs shape lure; color lure vs shape lure) and between trials with different RTs (e.g., slow- vs fast-RT trials).

### Data and code accessibility

All data and analysis scripts are publicly available in the OSF: https://osf.io/4yacu/.

## Results

Human observers performed two version of a visual-verification task that required verifying whether a visual stimulus on the screen matched either of two internal visual representations held in working memory (“mnemonic verification”; Fig. 1*a*) or whether a single visual representation held in working memory matched either of two external visual stimuli on the screen (“perceptual verification”; Fig. 4*a*).

Our primary focus was on tracking whether mnemonic verification engages refocusing (inspecting) specific mental representations for verification and to assess whether this occurs even when these representations are not actual targets but are perceived as potential targets. To this end, we defined targets by color–shape pairs but included “lures” (nontargets) that matched one of the memory objects only in color or only in shape (Fig. 1*a*; red and gray test types). Our rationale was the following: because lures look like potential targets, they may trigger internal inspection of similarly looking working-memory content to verify or dismiss a potential match.

To study such internal “in-mind” visual verification, we presented memory objects to the left and right while presenting the external comparison stimulus centrally (Fig. 1*a*). This enabled us to leverage spatial biases in fixational eye movements (microsaccades) to isolate internal attentional deployment within the spatial layout of working memory (as in van Ede et al., 2019, 2020, 2021; Draschkow et al., 2022; Liu et al., 2022; de Vries et al., 2023; Wang and van Ede, 2025). Our complementary perceptual-verification task (Fig. 4*a*)—which is more established (Downing, 2000; Olivers et al., 2006; Soto et al., 2008; Astle et al., 2009; Kuo et al., 2009; Dell’Acqua et al., 2010; Becker, 2011; Horstmann et al., 2016; Hamblin-Frohman and Becker, 2021)—served mainly as a reference for interpreting our key mnemonic-verification findings. We note how both tasks engage matching mnemonic with perceptual information but refer to these tasks as tracking “mnemonic” and “perceptual” verification, respectively, by virtue of our ability to experimentally isolate and track further visual inspection in working memory (mnemonic verification) or in perception (perceptual verification), as uniquely tagged by spatial location.

In what follows, we first provide eye-movement evidence for mnemonic (in-mind) verification that occurs not only for actual targets but also for lures that appear like potential targets. We show how such internal inspection occurs particularly when the verification is nontrivial and takes time. We next delineate similarities and differences between this uncovered mnemonic-verification behavior and the verification of potential perceptual targets, which is more established. Finally, we report EEG evidence for a common neural process mediating visual verification among the contents of working memory and among the contents of perception.

### Visual verification depends on perceived similarity between memorized and visible objects

Figure 1*b* shows behavioral performance on our mnemonic-verification task. While participants found it easy to dismiss shape lures (that always had a distinct color from the two memory objects) as nontargets, participants found it more difficult to reject color lures or to confirm actual targets. This was confirmed statistically. Repeated-measure ANOVAs revealed a significant main effect of the test type (target vs color lure vs shape lure) for both accuracy (*F*_(2,48)_ = 21.317; *p* < 0.001; $\eta_{p}^{2}=0.470$) and RT (*F*_(2,48)_ = 78.202; *p* < 0.001; $\eta_{p}^{2}=0.765$). Post hoc paired-sample *t* tests confirmed higher accuracy when the tested objects were shape lures, compared with color lures or actual targets (*t*_(24)_ = 5.875; *p* < 0.001; *t*_(24)_ = 5.351; *p* < 0.001, respectively) but also showed higher accuracy following color lures than following actual targets (*t*_(24)_ = 3.402; *p* = 0.007). For RT, we also confirmed that people found it easier to reject shape lures compared with color lures and actual targets (*t*_(24)_ = −12.167; *p* < 0.001; *t*_(24)_ = −8.604; *p* < 0.001, respectively), while color lures and actual targets did not differ (*t*_(24)_ = 1.419; *p* = 0.506). These effects cannot be attributed to participants’ bias toward particular test stimuli (particular shapes or particular colors), as specific shapes and colors were counterbalanced across conditions. Moreover, performance was comparable across all possible shapes and colors (Fig. S1).

While the observed lower accuracy for actual targets than for color lures may at least partially be explained by a response bias toward the “different” response option (given two-third of trials contained lures and only one-third targets), this cannot explain the observed differences between color and shape lures, as these lure types were always equally likely.

Together, these data show that while shape lures were easy to dismiss, any visual stimulus that matched the color of either visual representation in mind—an actual target or a color lure—was more difficult to arbitrate and engaged a process that took additional time, as evidenced by RT. As we show next, such color-matching stimuli called upon internal inspection of the color-matching memory object in service of arbitrating color lures from actual targets.

### Spatial saccade bias tracks internal inspection for potential visual matches

To track the inspection of internal visual memory objects in service of visual verification, we capitalized on our recent findings that have uncovered how attentional allocation within the spatial layout of visual working memory can be “read out” from spatial biases in fixational gaze behavior (van Ede et al., 2019, 2020, 2021; Draschkow et al., 2022; Liu et al., 2022; de Vries et al., 2023; Wang and van Ede, 2025), even in the presence of a large centrally presented stimulus (van Ede et al., 2021; de Vries et al., 2023; Wang and van Ede, 2025) as was also the case here. By presenting our memory objects to the left and right, but our test stimulus centrally (Fig. 1*a*), we could use such spatial biases to isolate internal attentional focusing, as a marker of mnemonic inspection associated with the internal verification process.

**Figure 2.**
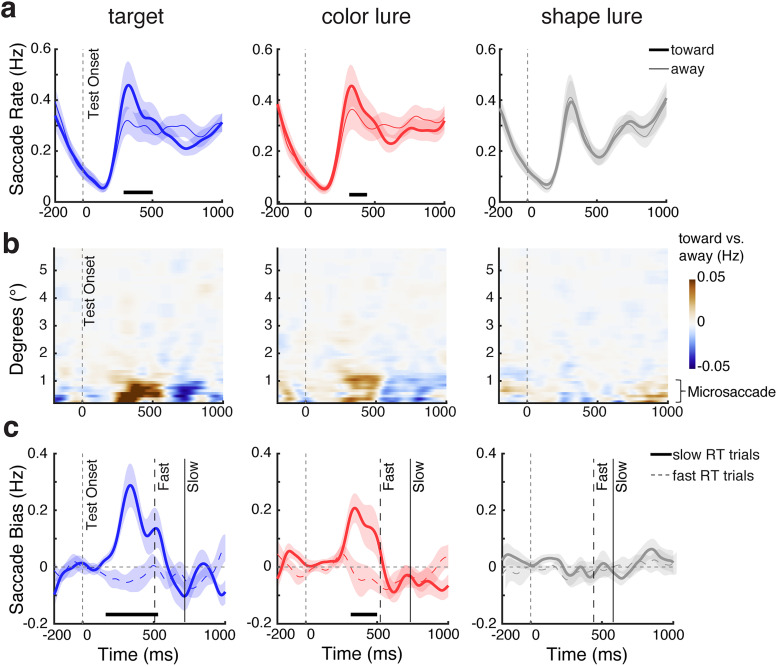
Spatial saccade bias tracks internal inspection following potential visual matches and is particularly pronounced when verification takes time. ***a***, Saccade rates (in hertz) separately for toward (bold lines) and away (thin lines) saccades relative to the original (encoded) location of the memory object that matched the central test stimulus, when the test was the actual target (left), color lure (middle), or shape lure (right). ***b***, Spatial saccade bias (the difference between toward and away saccades, with red colors denoting more toward saccades) as a function of the saccade size (*y* axis) and time (*x* axis) across test conditions. This shows how the spatial saccade bias was predominantly driven by saccades below 1 visual degree, consistent with a bias in microsaccades. ***c***, Spatial saccade bias (toward minus away) in trials with slow verification (RT > median RT; solid bold lines) and fast verification (RT < median RT; dashed thin lines) across test conditions (see the difference saccade bias between slow and fast-RT trials in Fig. S3*a*). The solid and dashed vertical lines indicate the median RTs (averaged across participants) in the depicted conditions. The thick black horizontal lines indicate significant difference clusters. Shadings indicate ±1 SEM across participants (*n* = 25).

**Figure 3.**
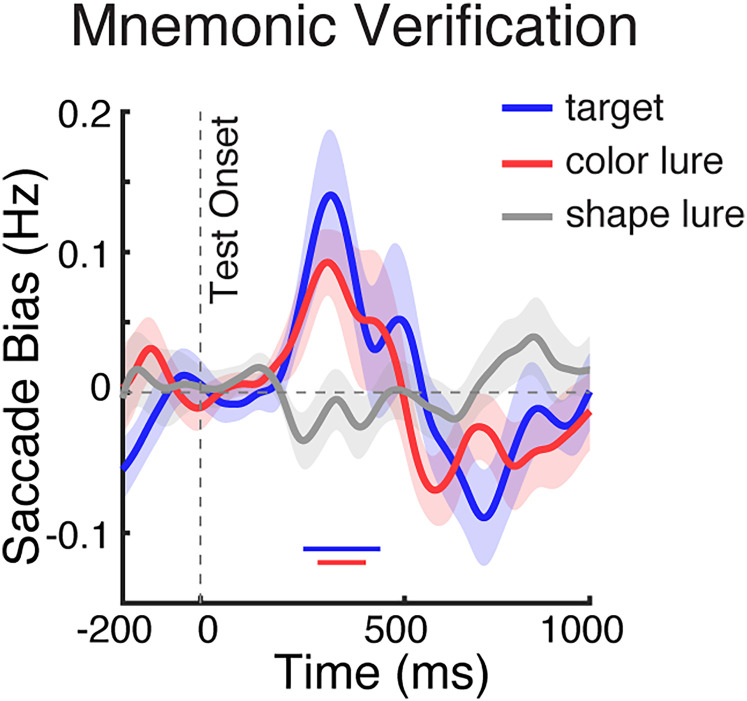
Spatial saccade bias tracks mnemonic inspection following potential visual matches. Overlay of spatial saccade bias (toward minus away, in hertz) in the mnemonic-verification task, across the three test conditions. Blue, red, and gray lines represent the gaze bias in response to actual targets, color lures, and shape lures. The blue, red, and gray horizontal lines above the *x* axis indicate significant clusters in each test condition, respectively (*p* < 0.05). Shadings indicate ±1 SEM across participants (*n* = 25).

Consistent with our behavioral results, we observed robust spatial saccade biases (Fig. 2) reflecting internal inspection following both targets and color lures, but not following shape lures that were less likely to be perceived (confused) as potential targets. Specifically, we found more saccades in the direction toward versus away from the location of the visual memory representation that matched the target (Fig. 2*a*, left; cluster *p* = 0.011) or whose color matched the color lure that was perceived as a potential target (Fig. 2*a*, middle; cluster *p* = 0.034). We present an overlay of the spatial saccade biases across our three conditions in Figure 3.

Consistent with our prior studies on this gaze marker of internal focusing (Liu et al., 2022; de Vries et al., 2023; Wang and van Ede, 2025), this saccade bias—quantified as the difference in rate between toward versus away saccades—was driven predominantly by small saccades within the classical microsaccades range (Fig. 2*b*). Moreover, this saccade bias was observed prior to reporting (Fig. 2*c*), consistent with an internal inspection process serving the ensuing report decision.

While we found clear evidence for internal attention shifts triggered by targets and color lures that were perceived as potential targets—thus prompting further inspection—we found no evidence for similar effects following shape lures (Fig. 2, right column). This likely reflects the fact that shape lures were easy to dismiss based on their unique color, without requiring further inspection. This is consistent with our behavioral data showing that participants rarely confused shape lures and were fast to dismiss them as nontargets.

Consistent with these primary eye-tracking results, we also found that the lateralization of posterior 8–12 Hz alpha-band activity tracked mnemonic verification in our target and color-lure conditions (Fig. S2), corroborating the internal inspection-for-verification process that we could track by virtue of having the memory items lateralized but the test-stimulus central.

### Internal inspection is particularly pronounced in slow-verification trials

We next reasoned that if the identified gaze bias reflects internal inspection in service of mnemonic verification, it should be particularly prominent in those trials where the additional verification is required but may come at the expense of additional time required for verification. In other words, it may be particularly pronounced in slow-verification trials. To address this prediction, we separated trials with fast and slow-verification decisions in our task, by performing a median split on RT within each condition. As shown in Figure 2*c*, this confirmed that the spatial gaze bias—as our marker of internal inspection—was pronounced particularly in those trials in which it took participants more time to verify whether the external stimulus matched the relevant internal representation or not. This was the case both when the stimulus was the actual target (Fig. 2*c*, left; slow vs fast cluster *p* < 0.001) or a color lure that was perceived as a potential target (Fig. 2*c*, middle; slow vs fast cluster *p* = 0.005). See also the difference saccade bias between slow and fast-RT trials in Figure S3*a*.

A comparison of response accuracy between slow- and fast-RT trials within each test condition in the mnemonic-verification task showed no statistically significant differences (|*t*|s ≤ 2.578; *p*s ≥ 0.181), suggesting slower responses were not due to lower accuracy.

To provide a more fine-grained link between our saccade-bias index of internal inspection and behavioral RT—and to further reveal the continuous nature of this relation—we also quartile-split the trials into four RT bins (Fig. S4). We observed a gradual increase in the strength of saccade bias with increasing response time. A one-way repeated–measure ANOVA with RT Bin (Bin 1 to Bin 4) as a within-subject factor on the mean saccade bias revealed a significant main effect of RT Bin in the target condition [*F*_(3,72)_ = 11.139; *p* < 0.001; $\eta_{p}^{2}=0.317$ (with a linear increasing trend, *F*_(1,24)_ = 23.191; *p* < 0.001; $\eta_{p}^{2}=0.491$] and in the color-lure condition [*F*_(3,72)_ = 7.804; *p* < 0.001; $\eta_{p}^{2}=0.245$ (with a linear increasing trend, *F*_(1,24)_ = 10.135; *p* = 0.004; $\eta_{p}^{2}=0.297$)], but not in the shape lure condition (*F*_(3,72)_ = 1.153; *p* = 0.334; $\eta_{p}^{2}=0.046$).

Finally, we also split the data into eight RT bins, which enabled us to run a linear regression between the size of the spatial saccade-bias (in the predefined 200–600 ms window; based on Liu et al., 2022; Wang and van Ede, 2025) and the mean RT in that bin. We did this for each participant and tested the resulting beta coefficients at the group level using one-sample *t* tests against zero. This confirmed a robust positive linear relation following actual targets (mean *β* = 0.542; *t*_(24)_ = 4.403; *p* < 0.001) and color lures (mean *β* = 0.578; *t*_(24)_ = 3.261; *p* = 0.003) but not shape lures (mean *β* = 0.053; *t*_(24)_ = 0.642; *p* = 0.527). Consistent with our median- and quartile-split analysis, this shows that the likelihood of saccades being directed toward (vs away from) the relevant memory object increased as visual verification following targets and color lures required more time.

We replicated these key eye-tracking findings in a second experiment that contained two additional conditions (as described in Text S1 and visualized in Fig. S6, as well as statistical results in Table S1).

For completeness, we also compared our saccade bias between correct- and incorrect- verification trials. Given that participants had generally high accuracy across all test conditions in the current experiment, the number of incorrect trials was small in each test condition (mnemonic verification task, 34.4, 15.2, 3.0 trials out of 864 trials in total). To increase statistical power, we collapsed incorrect trials across all three test conditions. This resulted in a total of 52.7 incorrect-response trials in the mnemonic-verification task, compared with 811.3 correct trials. As shown in Figure S5, the comparison of saccade bias between correct- and incorrect-response trials revealed a larger saccade bias for more difficult internal verifications, to the point that the bias was larger in trials when participants ended up making an incorrect-response (cluster *p* = 0.025, ranging from 442 to 547 ms after the test onset). These findings provide tentative evidence that complement our more robust RT-split–based results, corroborating our interpretation of stronger internal visual verification for visual targets that are more difficult to judge, even when these are ultimately misjudged. However, owing to the small number of trials and need to aggregate across test types, these results should be treated with caution and may (partly) reflect that in the conditions where we found larger gaze biases, there were also more incorrect trials.

### Mnemonic verification is similar but also distinct from perceptual verification

**Figure 4.**
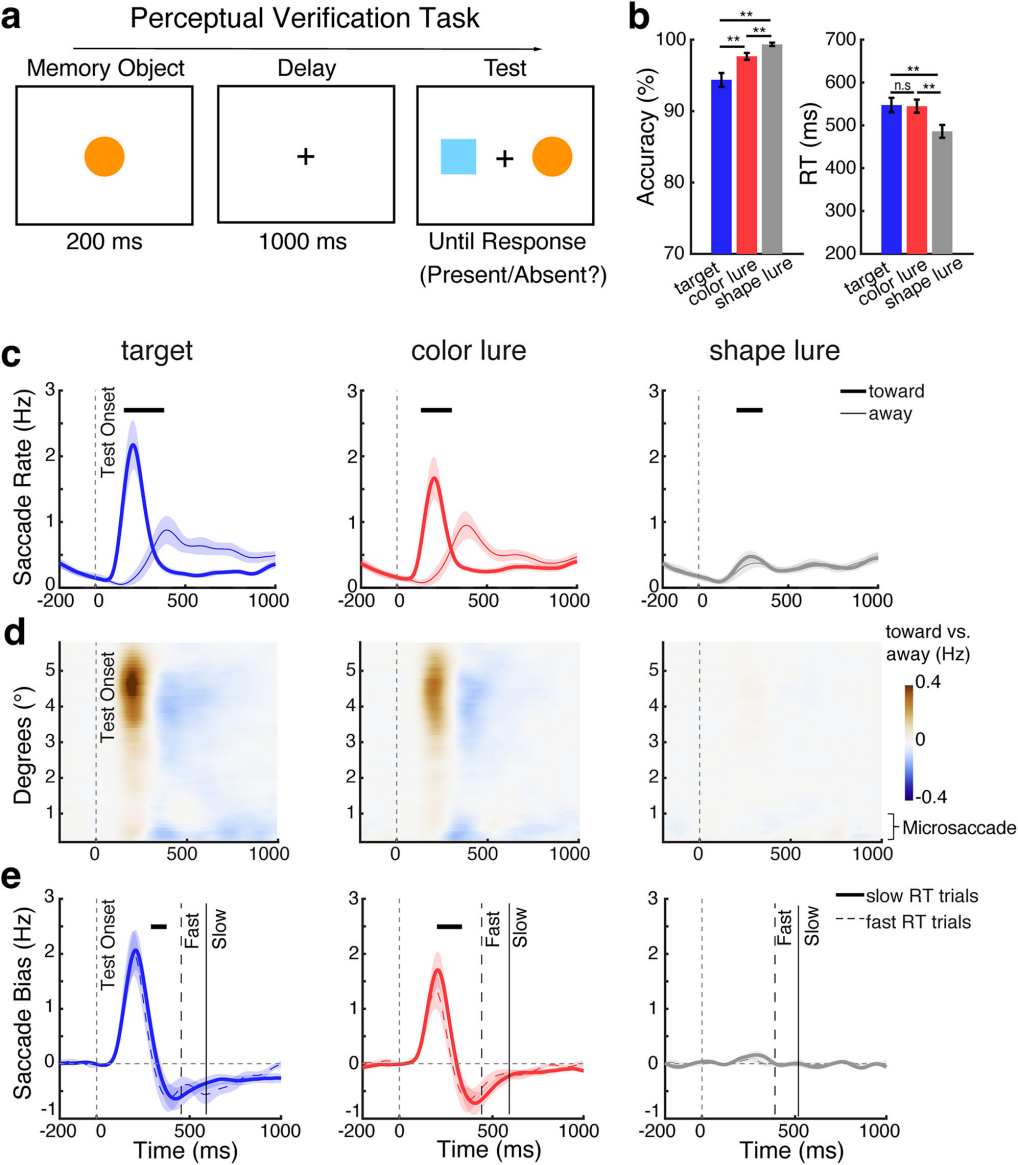
Task, behavior, and gaze in the perceptual-verification task. ***a***, Schematic of the perceptual-verification task. Participants memorized one centrally presented visual object and reported whether it was present or absent in a subsequent test array containing one left and one right visual stimulus. ***b***, Response accuracy (left panel) and response time (right panel) across test types. Error bars indicate ±1 SEM; ** represent *p* < 0.001; n.s represents not significant. ***c***, Saccade rates (in hertz) separately for toward (bold lines) and away (thin lines) saccades relative to the location of the test stimulus that matched the memorized object, when one of the test stimuli was the actual target (left), color lure (middle), or shape lure (right). ***d***, Spatial saccade bias (the difference between toward and away saccades, with red colors denoting more toward saccades) as a function of saccade size (*y* axis) and time (*x* axis) across test conditions. In this version of the task, the spatial saccade bias was driven by larger macrosaccades associated with looking at the matching external visual stimulus that was centered at 5.4°. ***e***, Spatial saccade bias (toward minus away) in trials with slow RT (RT > median RT; solid bold lines) and fast RT (RT < median RT; dashed thin lines) across test conditions (see the saccade-bias differences between slow and fast-RT trials in Fig. S3*b*). The solid and dashed vertical lines represent the median RTs (averaged across participants) in the depicted conditions. The thick black horizontal lines indicate significant difference clusters. Shading indicates ±1 SEM across participants (*n* = 25).

To relate our mnemonic-verification findings to the broader existing literature, we also examined behavioral and gaze patterns during a complementary perceptual-verification task (Fig. 4*a*) of which similar tasks have been used in ample prior research (Downing, 2000; Olivers et al., 2006; Soto et al., 2008; Kuo et al., 2009; Dell’Acqua et al., 2010; Eimer and Kiss, 2010; Töllner et al., 2014; Geng and Witkowski, 2019). While this task again required finding a match between internal and external visual stimuli, this time we used one central memory object and two potential perceptual targets that were now presented to the left and right. Accordingly, in this setup, spatial gaze biases signal perceptual inspection among the sensory contents of perception (rather than mnemonic inspection among the memorized contents of working memory as in the preceding results).

Similar to target verification in working memory, we again found that shape lures were easiest to dismiss, compared with color lures and actual targets (Fig. 4*b*). This was confirmed statistically. Repeated-measure ANOVAs revealed significant main effect of the test type for both accuracy (*F*_(2,48)_ = 27.843; *p* < 0.001; *η*^2^_p_ = 0.537) and RT (*F*_(2,48)_ = 43.447; *p* < 0.001; *η*^2^_p_ = 0.644). Post hoc paired-sample *t* tests confirmed higher accuracy when the tested objects were shape lures, compared with color lures or actual targets (*t*_(24)_ = 4.218; *p* = 0.001; *t*_(24)_ = 5.588; *p* < 0.001, respectively) but also showed higher accuracy following color lures than following actual targets (*t*_(24)_ = 5.032; *p* = 0.007). For RT, we also confirmed that participants found it easier to reject shape lures compared with color lures and actual targets (*t*_(24)_ = −13.219; *p* < 0.001; *t*_(24)_ = −6.722; *p* < 0.001, respectively), while color lures and actual targets did not differ (*t*_(24)_ = −0.338; *p* = 1.000).

**Figure 5.**
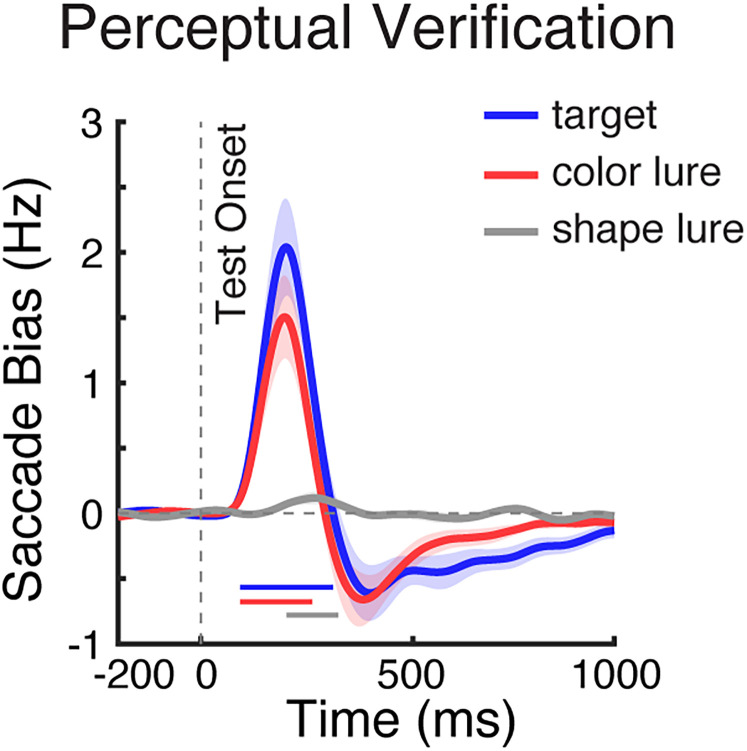
Spatial saccade bias tracks perceptual inspection. Overlay of spatial saccade bias (toward minus away, in hertz) in the perceptual-verification task, across the three test conditions. Blue, red, and gray lines represent the gaze bias in response to actual targets, color lures, and shape lures. The blue, red, and gray horizontal lines above the *x* axis indicate significant clusters in each test condition, respectively (*p* < 0.05). Shadings indicate ±1 SEM across participants (*n* = 25).

When considering gaze, we also replicated clear spatial saccade biases to targets as well as to lures—whose locations were now not in working memory but on the screen (Fig. 4*c*). While this was again clearest for actual targets (cluster *p* = 0.006) and color lures (cluster *p* = 0.005), the bias also reached significance for shape lures (cluster *p* = 0.024). An overlay of the spatial saccade biases between conditions can be found in Figure 5. Unlike during mnemonic verification, where we found a spatial bias in the microsaccade range, during perceptual verification, this bias was driven by larger (regular) “macro” saccades (Fig. 4*d*), consistent with the fact that now there were external visual stimuli (rather than internal memory representations) to inspect. We further replicated a relation between the magnitude of this bias and participant's RT (Fig. 4*e*) following actual targets (Fig. 4*e*, left; slow vs fast cluster *p* = 0.004) and color lures (Fig. 4*e*, middle; slow vs fast cluster *p* < 0.001); see also the difference saccade bias between slow and fast-RT trials in Figure S3*b*, again showing a larger bias in slow-verification trials. However, unlike what we observed for mnemonic inspection, perceptual inspection was pronounced even when participants were fast and did not differ significantly between correct- and incorrect-response trials (Fig. S5*b*).

**Figure 6.**
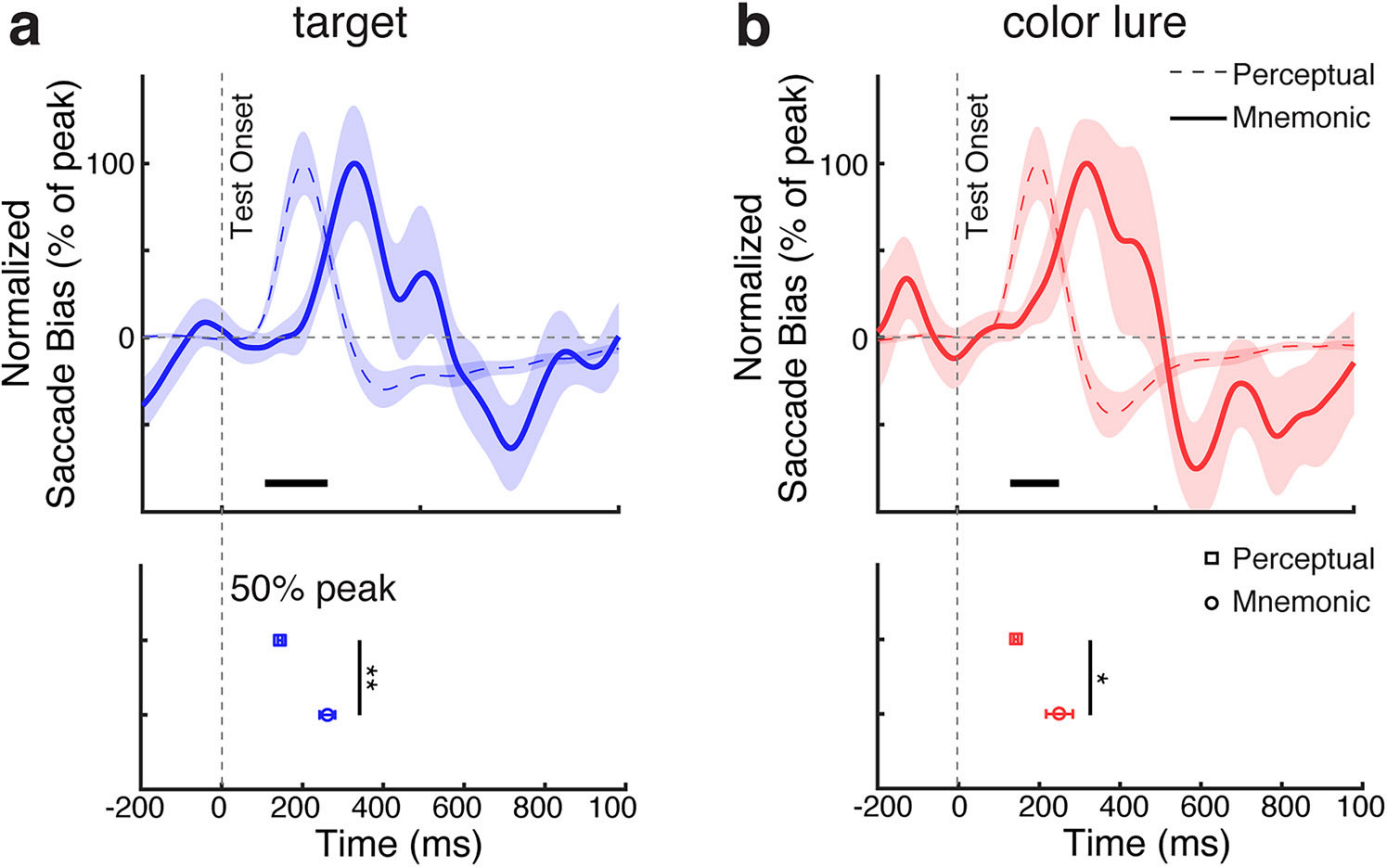
Mnemonic inspection is initiated slower than perceptual inspection in service of visual verification. Top panels, Normalized spatial saccade biases (toward vs away) associated with mnemonic inspection (solid bold lines) and perceptual inspection (dashed thin lines) following onset of (***a***) actual targets and (***b***) color lures. The thick black horizontal lines indicate significant difference clusters. Shading and error bars indicate ±1 SEM across participants (*n* = 25). To facilitate comparison between mnemonic and perceptual-verification processes, saccade-bias time courses were normalized to their peak value. Bottom panels, Onset latency of the spatial gaze bias, defined as the first sample reaching 50% of the peak, during perceptual (squares) and mnemonic (circles) verification. ** and * represent *p* < 0.01 and *p* < 0.05, respectively.

To further elucidate the differences between visual target verification by further inspecting working memory or perception, we directly compared the gaze patterns between the mnemonic and perceptual tasks versions. Comparing the normalized saccade bias (peak normalized to be in the same range), we found that the saccade bias started substantially later during mnemonic inspection among the contents of working memory compared with during perceptual inspection among the contents of perception, following both actual targets and color lures (Fig. 6*a*,*b*). This was confirmed statistically using a jackknife test on the onset latency (quantified as the first value reaching 50% of the peak) both following actual targets (Fig. 6*a*; 261.6 ms for mnemonic inspection vs 144.5 ms for perceptual inspection, *p* < 0.001) and following color lures (Fig. 6*b*; color lure, 250.2 ms for mnemonic inspection vs 142.0 ms for perceptual inspection, *p* = 0.005).

### Frontal midline theta activity tracks both mnemonic and perceptual verification

Our behavioral and gaze data suggested robust visual inspection processes following actual targets and color lures, but not following shape lures. Presumably, in our task, shape lures could be dismissed without further inspection, purely based on their distinctive color. Here, we leveraged this observation to investigate the neural dynamic of the inspection-for-verification process using EEG. The relative lack of a putative inspection-for-verification process in the shape lure condition enabled us to use this condition as a reference condition to contrast to the other two conditions that did show clear evidence of such verification processes.

**Figure 7.**
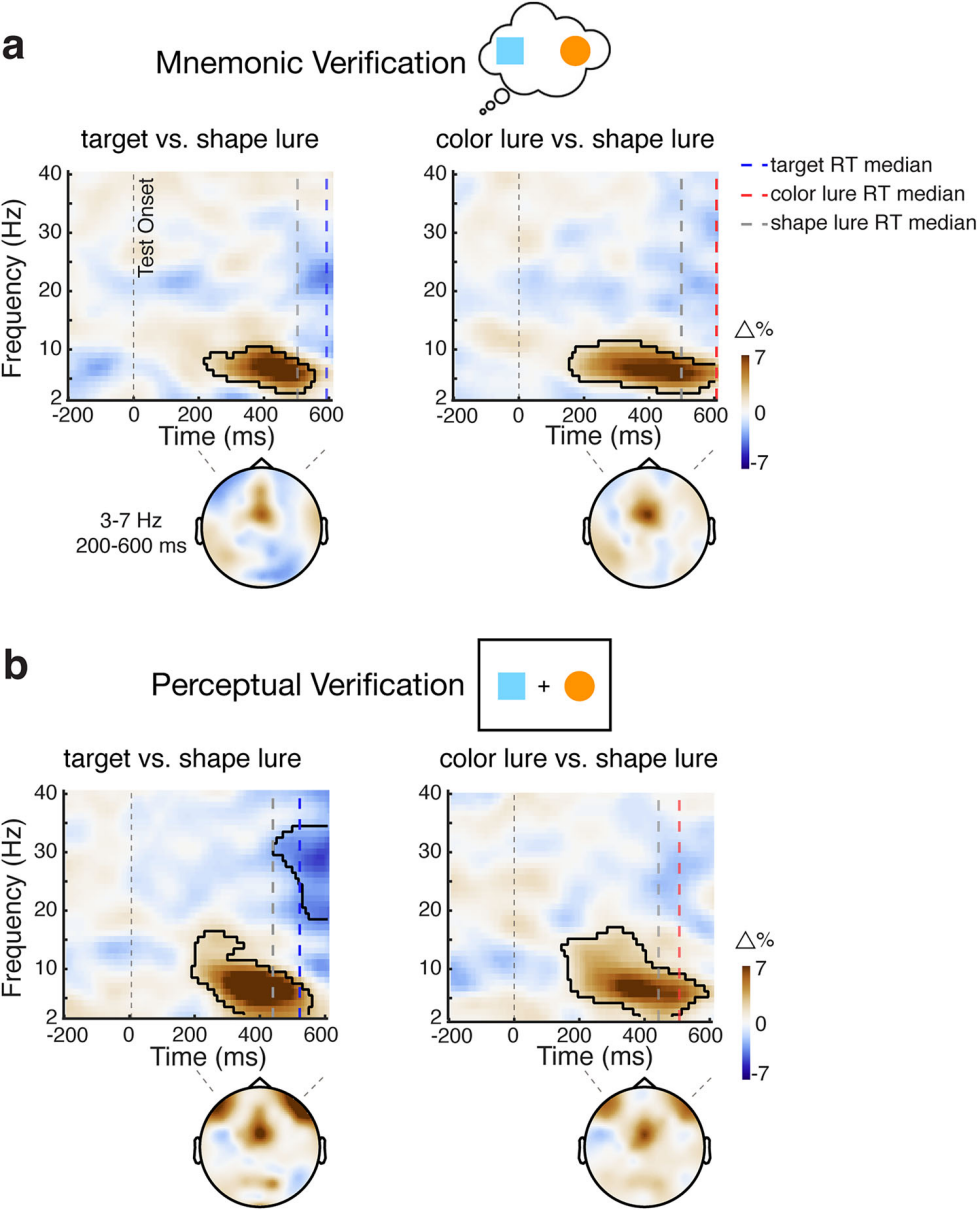
Frontal midline theta activity tracks visual verification among the contents of working memory and perception. ***a***, Mnemonic verification, Time–frequency maps of the percentage change in spectral power following targets (left panels) and color lures (right panels) in comparison with shape lures that here served as the neutral reference condition. The colored vertical dashed lines represent median RT for each condition (averaged across participants). Blue, red, and gray lines represent median RT for target, color lure, and shape lure, respectively. Black outlines indicate significant clusters. Topographies show the difference in power averaged over 3–7 Hz and 200–600 ms after the test onset. ***b***, The same conventions as in panel ***a*** but for the data from the perceptual-verification task.

This revealed a relative increase in frontal midline theta (∼3–7 Hz) activity in the two conditions that showed the visual inspection-for-verification process of interest. This was the case during both mnemonic verification (Fig. 7*a*; cluster *p* = 0.035 and 0.003 for the target and color-lure conditions, respectively) and perceptual verification (Fig. 7*b*; cluster *p* = 0.006 and 0.005 for the target and color-lure conditions, respectively). This frontal theta increase happened before response onset (see median condition-specific RTs indicated by the vertical dashed lines in Fig. 7*a*,*b*), consistent with reflecting the verification process preceding the report.

**Figure 8.**
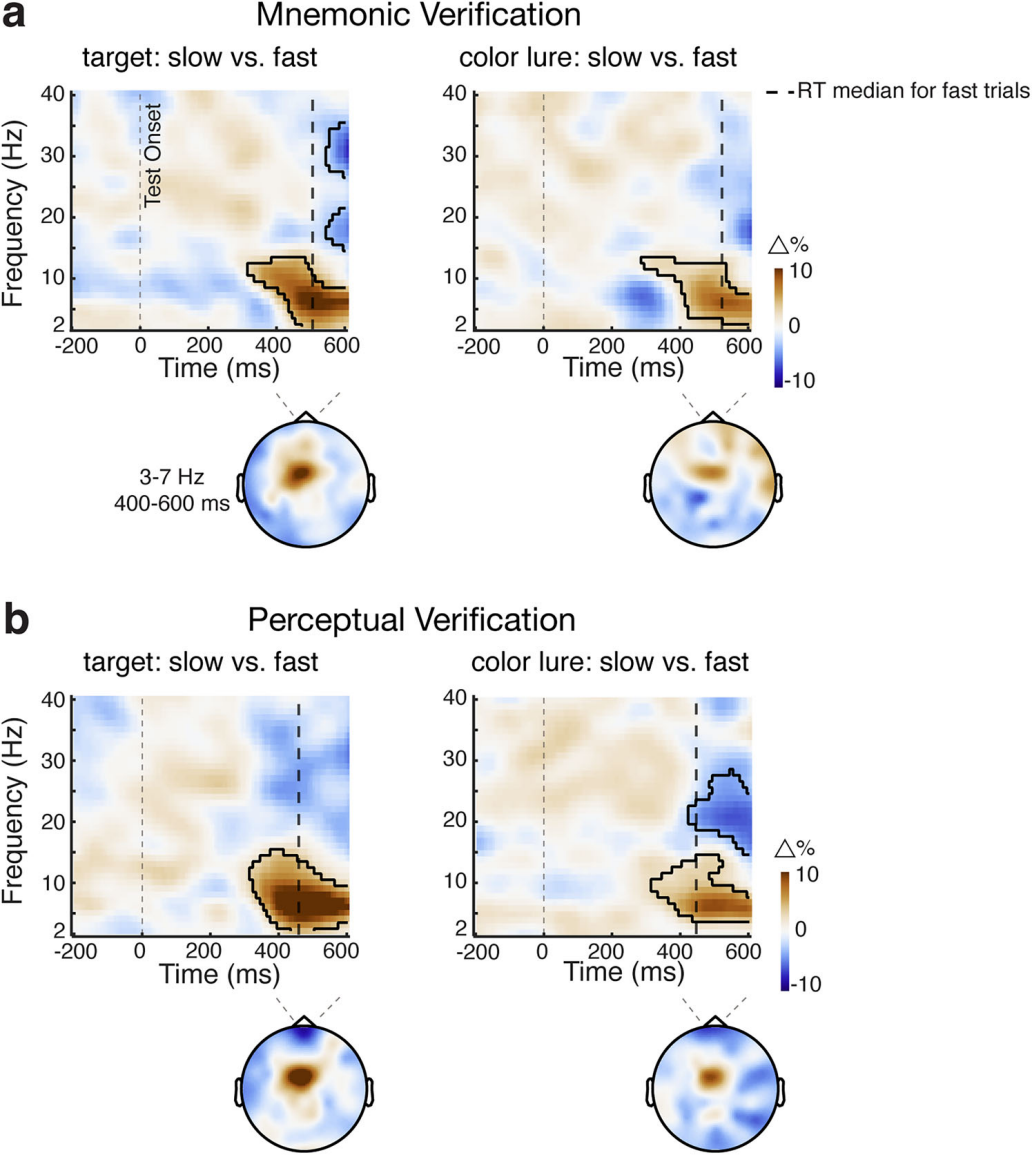
Frontal midline theta activity is particularly pronounced when verification takes time. ***a***, Mnemonic verification, Time–frequency maps of the percentage change in spectral power between slow minus fast-verification trials (separated by a median split), within the target (left panels) and color lure (right panels) verification conditions of interest. The black vertical dashed lines represent median RT for fast-verification trials for each condition (averaged across participants). Black outlines indicate significant clusters. Topographies show the difference in power averaged over 3–7 Hz and 400–600 ms after the test onset. ***b***, The same conventions as in panel ***a*** but for the data from the perceptual-verification task.

Additionally, our data in Figures 2*c*, 4*e* suggested that this verification process (in the target and color-lure conditions) was particularly pronounced in trials with slow responses where, presumably, the verification process was more demanding and thus time-consuming. Accordingly, if frontal theta activity supports the verification process, it should also be more pronounced in slow trials. This is precisely what we found, with stronger frontal theta activity in slow-RT compared with fast-RT trials (Fig. 8*a*; mnemonic verification, cluster *p* = 0.024 and 0.028 for the target and color-lure conditions, respectively; Fig. 8*b*; perceptual verification, cluster *p* < 0.001 and 0.004 for the target and color-lure conditions, respectively).

## Discussion

Models of visual search—finding what we are looking for—naturally invoke the notion of a comparison between internal memory representations (search goals) and incoming external sensory information (Hyun et al., 2009; Carlisle et al., 2011; Wolfe and Horowitz, 2017; Wolfe, 2021). Accordingly, visual verification to confirm or dismiss a potential visual match may not only call upon inspection among the external contents of perception (as in Olivers et al., 2006; Soto et al., 2008; Hollingworth and Luck, 2009; Geng and Witkowski, 2019) but also among the internal contents of working memory. Yet, studies directly tracking the latter remain scarce and have left key questions unaddressed. Here, we provide evidence that humans “look back” into working memory during visual search. Consistent with a verification process, we show that such mnemonic inspection occurs not only when a visual target matches a mnemonic search goal but also when it resembles a potential search goal that warrants further internal inspection. We further show how this process scales with observed verification behavior, being particularly pronounced when it takes more time to resolve a potential match. These findings underscore the relevance of internal verification processes in visual search and provide new ways to expose and track this underexplored component of visual search.

At least two prior event-related potentials studies have reported how finding a match between visual information in mind and world can engage shifts of attention to matching internal representations within the spatial layout of visual working memory (Kuo et al., 2009; Dell’Acqua et al., 2010; for a complementary behavioral demonstration, see also Theeuwes et al., 2011). Our findings build on and extend this work in three ways. First, we show that such mnemonic inspection also occurs for potential targets that merely resemble what we are looking for. Because aforementioned studies only tracked internal inspection following match targets, prior findings could have reflected the verification process itself (inspection serving verification) or the consequence of having found a match. Our study now confirms the former interpretation. Second, we relate this internal inspection process to observed verification behavior, showing it is particularly pronounced when the verification process requires more time. The more pronounced verification in slow trials likely reflects that this process is called upon particularly when the comparison is more challenging. Observed condition differences may also reflect differences in difficulty and/or verbalization of memorized content. At the same time, stronger saccade bias for slower-RT trials was observed only in the actual-target and color-lure conditions, not in the shape lure condition where participants could easily dismiss the lure based on its distinctive color, without requiring further internal inspection. This suggests that our gaze marker does not generally scale with RT but only when there is an internal inspection process that takes time. Finally, our data showcase the usefulness of gaze as a powerful complementary measure to track internal attentional focusing during search that we here interpret as a mnemonic inspection process serving verification. Such “looking into memory” may benefit visual verification by targeted inspection of the diagnostic feature (shape) of the resembling (color-matching) memory object. Moreover, by attending the resembling memory object, this object may also be placed in a prioritized state that may further benefit search by facilitating external visual guidance (Olivers et al., 2011).

In the current study, we introduced color and shape lures to investigate the mnemonic-verification process and found that it was particularly evident following color lures. We were able to leverage this asymmetry by using the shape lures as a neutral baseline for our EEG comparisons. However, we do not claim that the reported mnemonic-verification process occurs exclusively when potential targets match in color but not in shape. Ultimately, this likely depends on the nature of the task and stimuli. In our task, color was likely more distinguishable and therefore served as a more diagnostic feature than shape (cf. Boettcher et al., 2016; Biderman et al., 2017; Geng et al., 2017; Niklaus et al., 2017; Geng and Witkowski, 2019). When the external stimulus had a different color from the memory representations (shape lure), it could easily be dismissed without prompting further mnemonic inspection. However, when the external stimulus had the same color as one of the memory objects (color lure), it prompted additional inspection, to determine whether it also had the same shape. In future studies, it will be interesting to parametrically vary the diagnostic values of shape and color (and other potential visual features). We predict that mnemonic inspection is triggered by perceived ambiguity, regardless of whether this ambiguity is driven by shape, color, or other features.

We found significantly lower response accuracy in the actual-target condition compared with the two nontarget-lure conditions. This might be driven by participants’ bias toward making a nonmatch response, given that two-thirds of the trials were nonmatches. We intentionally designed the experiment this way to balance the number of trials across the three test conditions, thereby making the eye-tracking and EEG variables comparable across conditions. Moreover, our main evidence linking internal inspection to behavioral performance comes from within-condition comparisons (i.e., fast-RT vs slow-RT trials), for which we exclusively considered correct trials. Nevertheless, our study leaves unadressed whether results would differ when this response bias is controlled (e.g., by equalizing the number of match and nonmatch trials). Future work is also required to understand the complementary processes that ultimately lead to a confirmation versus dismissal of potential targets. Our data provide only partial insight into this complementary question, showing a lack of internal inspection when objects are easy to dismiss based on their unique colors.

A direct comparison between the understudied internal verification process and the widely studied perceptual-verification process revealed important commonalities and differences. Verification processes in both modalities were associated with frontal midline theta activity that was more profound when the verification process took more time. This aligns with the well-established role of midline frontal theta activity in cognitive control and working memory (Jensen and Tesche, 2002; Cavanagh and Frank, 2014; van Ede et al., 2017; Cooper et al., 2019; de Vries et al., 2019; Chidharom and Carlisle, 2023; Wen et al., 2023; Ding et al., 2024). At the same time, we observed notable differences. First, while mnemonic verification was predominantly reflected in microsaccades, perceptual verification was evident in larger saccades, consistent with the overt inspection of visual stimuli on the screen. We interpret these microsaccade biases as an overt reflection (peripheral fingerprint; Corneil and Munoz, 2014) of covert attention shifts within the spatial layout of working memory (as in van Ede et al., 2019, 2020, 2021; Draschkow et al., 2022; Liu et al., 2022; de Vries et al., 2023; Wang and van Ede, 2024, 2025). We further found a significant difference in timings. Inspection among the contents of working memory commenced later than inspection among the contents of perception in our task. This slower inspection in the mnemonic-verification task may be (at least partly) driven by its relatively higher difficulty compared with the perceptual-verification task.

Despite us treating mnemonic and perceptual verification as separate, ultimately the comparison between external visual targets and internal memory representations relies on a two-way process that engages joint “mnemonic” plus “perceptual” verification. Here, we experimentally isolated the mnemonic from the perceptual-verification components by selectively studying visual inspection among the contents of working memory (“mnemonic verification”) or perception (“perceptual verification”). We did this by selectively tagging either the memory or the perceptual objects-to-inspect to specific spatial locations (adopting the same logic as in Kuo et al., 2009; Dell’Acqua et al., 2010). Yet, in real-world search, the processes of internal and external inspection in service of verification are likely often engaged concurrently and may interact in interesting, yet to be understood, ways. In future studies, it will be interesting to develop tasks to track both subprocesses concurrently. We further point out that while our study specifically targeted search from working memory, search may also be guided by long-term memory, where external and internal inspection likely also work together (Woodman and Chun, 2006; Wolfe, 2012; Wolfe et al., 2015).

Our experimental task and analysis approach open new avenues for studying core components of visual search that are often indirectly inferred but rarely directly tracked. By positioning memory objects in distinct locations from the visual search object, we could leverage directional biases in microsaccades to isolate and track mnemonic inspection during visual search. Moreover, by including lures, we could track internal inspection not only when faced with actual targets but also in response to external stimuli that were perceived as potential targets and thus warranted further inspection. This approach could be expanded to study internal verification processes in more complex and elaborate search paradigms, such as hybrid search (Cunningham and Wolfe, 2014; Drew et al., 2016). This has ample potential to provide additional insights into the foundational two-way processes by which we identify what we are looking for and dismiss what we are not.
